# Optical metasurfaces for high angle steering at visible wavelengths

**DOI:** 10.1038/s41598-017-02167-4

**Published:** 2017-05-23

**Authors:** Dianmin Lin, Mauro Melli, Evgeni Poliakov, Pierre St. Hilaire, Scott Dhuey, Christophe Peroz, Stefano Cabrini, Mark Brongersma, Michael Klug

**Affiliations:** 1 Magic Leap Inc., Plantation, FL 33322 USA; 2 The Molecular Foundry, Lawrence Berkeley National Laboratory, Berkeley, CA 94720 USA; 3 Geballe Laboratory for Advanced Materials, Stanford University, Stanford, CA 94305 USA

## Abstract

Metasurfaces have facilitated the replacement of conventional optical elements with ultrathin and planar photonic structures. Previous designs of metasurfaces were limited to small deflection angles and small ranges of the angle of incidence. Here, we have created two types of Si-based metasurfaces to steer visible light to a large deflection angle. These structures exhibit high diffraction efficiencies over a broad range of angles of incidence. We have demonstrated metasurfaces working both in transmission and reflection modes based on conventional thin film silicon processes that are suitable for the large-scale fabrication of high-performance devices.

## Introduction

Optical metamaterials are gaining increased attention for their ability to control light beyond the capabilities of conventional optics^1–12^. In particular, the concept of a metasurface - a two-dimensional metamaterial - holds the promise of replacing bulky optical components with ultrathin planar photonic structures^1, 9, 13–17^. Gradient metasurfaces are composed of nanoscale optical resonators - also called antennas - capable of manipulating light by imparting local phase changes on incident electromagnetic waves^17–21^. Moreover, metasurfaces afford control over the polarization, phase and amplitude of the light, thus opening the door to create new optical functionalities^22–24^. Ultrathin and planar optical elements based on metasurfaces have been realized for light focusing or redirecting^13, 15, 25–28^, photonic spin control^8, 23, 29, 30^, holograms generation^31, 32^, nonlinear light concentration^10^ and actively tuning of the optical properties^33^.

Recently, to develop ultra-compact beam control elements, metasurfaces with linear phase profiles that are based on dielectric and metallic resonators have been used^1, 9, 34, 35^. These metasurface-based optical elements can be easily integrated into a variety of systems due to their ultrathin and flat nature. Metasurfaces are also much easier to fabricate than the traditionally used saw-tooth or blazed gratings using already available fabrication techniques. In previous designs of planar effectively-blazed metasurfaces, the grating period was large (3.2 μm) thus limiting the deflection angles to ~8° at a wavelength of 550 nm^9^. A grating period at the sub-wavelength scale is required in order to achieve high angle steering. It is challenging to reduce the period of the grating because optically resonant antennas are typically a non-negligible fraction of the free-space wavelength and high diffraction efficiencies can only be achieved by having many antennas per grating period^13^. For example, high-efficiency designs typically feature eight optical antennas (for eight discrete phase levels) within one lattice period^13^. Also, when the grating period is smaller than the wavelength, the diffraction efficiency of a blazed grating significantly drops due to the shadow effect, even when implemented as a planar metasurface^4, 36^. In addition, the absorption of popular high-index dielectric material in the visible spectrum is typically high, and so many high-efficiency metasurfaces have targeted the infrared spectrum^22, 35^. All of these factors make it challenging to design metasurfaces that efficiently steer light to large deflection angles at visible wavelengths. Additionally, these metasurfaces have primarily demonstrated functionality with plane waves at a normal incidence; its diffraction efficiency dramatically decreases upon illumination with off-normal incidence angles. Using optical elements for wide angle steering with a large acceptance angle at visible wavelengths is unprecedented in the literature. A large range of acceptance angles, and uniform efficiency across the angular range, would be desirable for optical elements in many applications.

Here, we present two types of metasurface implementations of blazed gratings that are able to efficiently steer light in the visible range with a relatively uniform efficiency over a wide range of incidence angles. One design works in transmission mode and the other in reflection mode. Both structures consist of a periodic arrangement of double sub-wavelength silicon nanobeams. In addition, our metasurfaces exhibit strong polarization dependence and therefore can act as polarizing beam splitters.

## Results

### Design principles of metasurfaces for high angle steering

**Figure 1:**
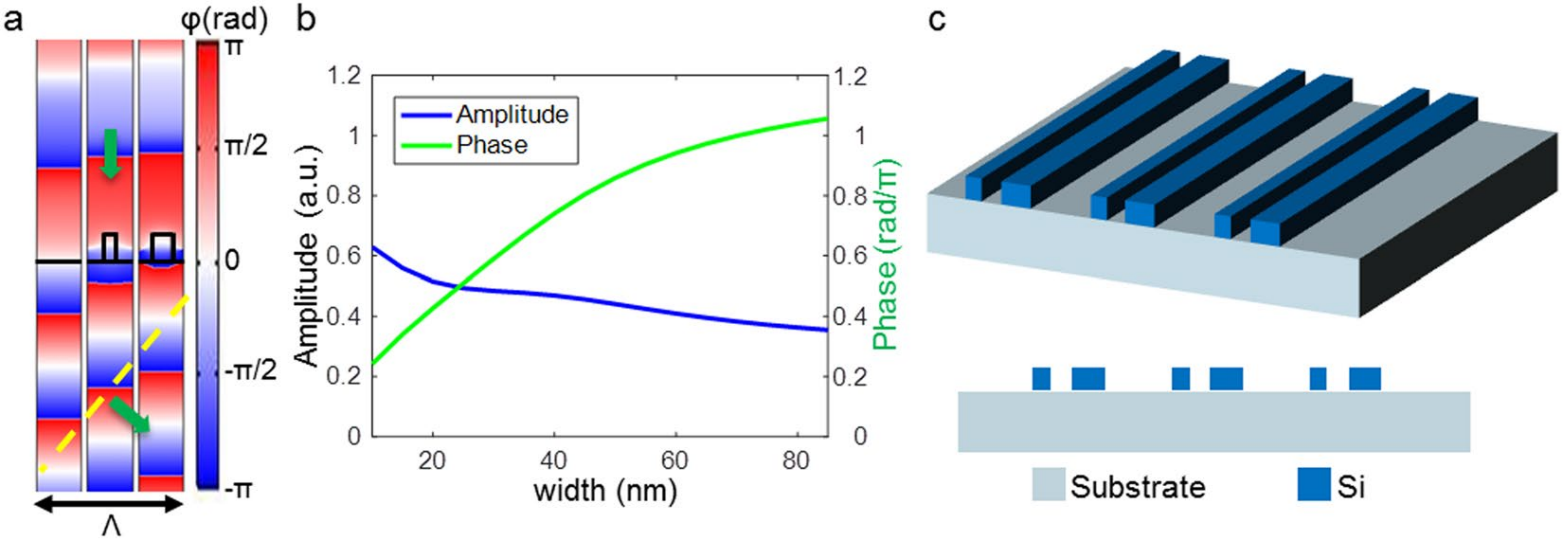
Design of transmission mode metasurfaces with a large steering angle. (**a**) Phase wavefronts of the TM-polarized transmission mode metasurface at a 520-nm wavelength without the Si nanobeams, and with an array of nanobeams of 30 nm and 55 nm widths respectively spaced by 125 nm. (**b**) The variation of the phase delay and amplitude of the transmitted light versus the width of the Si nanobeams. (**c**) Schematic of the designed metasurface, which consists of two nanobeams with a thickness of 75 nm and widths of 30 nm and 55 nm, with an edge-to-edge gap of 85 nm and periodicity of 380 nm. The bottom inset shows the side view of the metasurface.

Nanobeams composed of a high refractive index semiconductor material exhibit strong electrical and magnetic optical antenna resonances^37–43^. With the nanobeams arranged with sub-wavelength spacing, the optical resonance persists, and only zero-order light is transmitted. The phase delay of the transmitted light through the array of nanobeams can be controlled by the thickness and width of the nanobeams. The wavefront of transmitted light with a transverse magnetic (TM) polarization (with the electric field parallel to the nanobeams) at a wavelength of 520 nm is shown in Fig. 1a. For a periodic array consisting of 30-nm-wide, 75-nm-thick Si nanobeams spaced by 125 nm, finite element simulations indicate that the wavefront of transmitted light is delayed compared to the wavefront in the absence of the nanobeams. The wavefront for larger nanobeams of 55 nm width is delayed even further, which can be understood from the effective index theory and optical resonances. As a result, by engineering the size of the nanobeams, the phase of transmitted light can be modulated. Figure 1b shows the variation of the phase and amplitude of light transmitted through metasurfaces versus the width of nanobeams made of Si. Although it is shown that the range of phase pickup by the nanobeams is less than 2π, a tilted phase wavefront with a large phase gradient can be produced when nanobeams with different widths are arranged at deep sub-wavelength spacing and the grating pitch is smaller than the wavelength of light. The grating of high spatial frequency that we are demonstrating here constrains us from having more than three phase levels within one grating pitch. Consequently, the maximum phase modulation that is required for a three-level phase approximation of a blazed grating is less than 2π, while the diffraction efficiency will be reduced when compared to gratings of lower spatial frequency^13^. The amplitude of the transmitted beam remains relatively flat across different widths of nanobeams but is attenuated with the increasing width of Si nanobeams due to absorption.

The optical response of the nanobeam array is robust for different incidence angles, which can largely be understood by understanding the scattering properties of the individual, constituent Si nanobeams. Each nanobeam can be considered as a slab waveguide that has been truncated in the propagation direction. The acceptance angle of a slab waveguide is determined by the refractive index of its core material and cladding material. Here, each nanobeam has a large acceptance angle thanks to the high refractive index contrast between silicon and air. In addition, the silicon nanobeams are subwavelength in thickness, which reduces the shadow effect from neighboring antennas. It is shown that the phase delay and amplitude of the nanombeam array upon tilt angle of incidence is close to that of normal incidence (see Supplementary Fig. S1).

By utilizing these Si nanobeams, we have created metasurfaces with a high tilted phase profile capable of steering incident light to very large deflection angles with high diffraction efficiency over a wide range of acceptance angles. Figure 1c presents a schematic of the designed metasurface that is functional in transmission mode. The metasurfaces are constructed by placing three distinct elements into a repeating unit cell: an interval without a nanobeam followed by two differently sized nanobeams, which form a three-level discretization of a quasi-linear phase profile. The nanobeams have a thickness of 75 nm and width of 30 nm and 55 nm with an edge-to-edge gap of 85 nm. The diffraction efficiency in the proposed design is optimized to be as high as possible across the entire angular range of incidence angles. In the optimization process, the thickness of the nanobeams is adjusted to balance covering a large range of phase modulation while at the same time retaining optical properties that are less sensitive to the angle of incidence. The widths of nanobeams and gaps between nanobeams are optimized to provide a constant efficiency across the full range of incidence angles. The pitch size of a unit cell is designed to be 380 nm, which gives rise to a deflection angle of 50 degrees upon normal incidence at a wavelength of 520 nm. The deflection angle follows the grating equation and varies with incidence angle and wavelength, which are plotted in Supplementary Figs S2 and S3 respectively.

**Figure 2:**
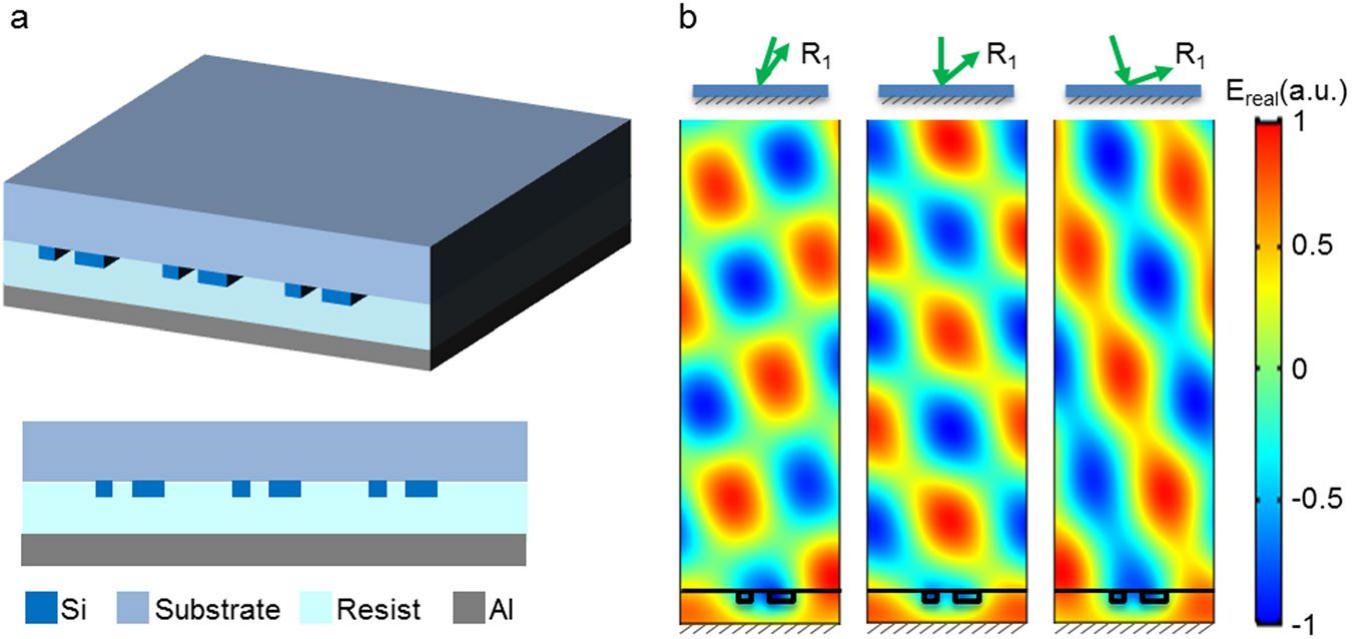
Design of reflection mode metasurfaces. (**a**) Metasurface schematic with a PMMA spacer layer between the nanobeams and the metal layer. The nanobeams have a thickness of 25 nm and widths of 30 nm and 60 nm respectively, with an edge-to-edge gap of 45 nm and periodicity of 380 nm. (**b**) Simulated total out-of-plane scattered electric field at a wavelength of 520 nm at incidence angles of −18, 0 and 18 degrees. The insets above the electric field maps show the schematic diagram of the +1 order of diffraction from the reflection mode metasurface. The green arrows indicate the wave vectors of the incident light and the +1 reflected order of diffraction.

We have also designed analogous functional metasurfaces in reflection mode. Figure 2a shows the schematic of a metasurface working in reflection mode. In reflection mode, the light interacts with the nanobeams twice (once incident and again after reflection off the back mirror), which gives rise to a higher diffraction efficiency (see Supplementary Fig. S4). The design principle of the reflection mode metasurface is similar to the transmission case: two nanobeams with different widths and spacing apply a tilted phase retardation of the wavefront. The grating period that determines the diffraction angle is the same as in the transmission mode case. The metasurface optimized for an operational wavelength of 520 nm is constructed from two nanobeams with widths of 30 nm and 60 nm and an edge-to-edge spacing of 45 nm. The thickness of the nanobeams is only 25 nm, much thinner than in the transmission mode case, since the light interacts twice with the metasurface. In the reflection case, the nanobeams are coated with a polymer of refractive index n = 1.45 (PMMA) and are followed by a layer of metallic film. The low refractive index polymer acts as a spacer and its thickness is carefully optimized to reach a high diffraction efficiency. The interference between the reflection from the metasurface and the metal film produces a high efficiency in the first diffraction order and low diffraction efficiency in 0^th^ order reflection. Figure 2b shows the out-of-plane scattered electric field distribution (real part of complex) at wavelength of 520 nm upon a wide range of incidence angles. The incident light is subtracted out in the field plot, showing only the scattered fields. There are interference patterns for the reflection wavefronts due to the interference between −1 and +1 reflected order.

### Experimental demonstrations and numerical simulations

**Figure 3:**
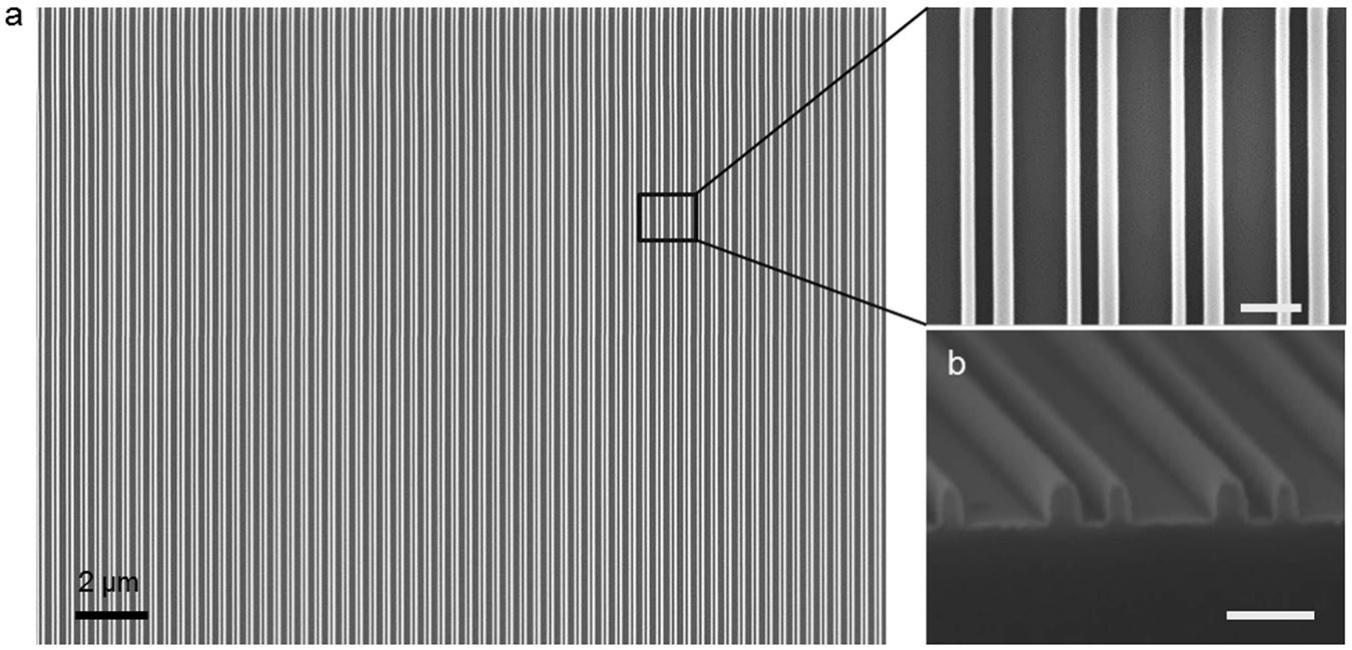
Scanning electron microscope images of the transmission mode metasurface. (**a**) SEM from the top view and with zoom-in image. (**b**) SEM of the cross section of the structure. The scale bar for the images in the right column is 200 nm.

To experimentally verify the possibility of realizing metasurfaces for high-angle steering, we fabricated the metasurface described above using the nanofabrication techniques presented in the methods section. Figure 3 shows scanning electron microscopy (SEM) images of the fabricated transmission mode metasurface. Here electron beam lithography is used for nanopatterning for proof of concept. For mass production, other parallel lithography techniques could be employed for large-area nanopatterning.

**Figure 4:**
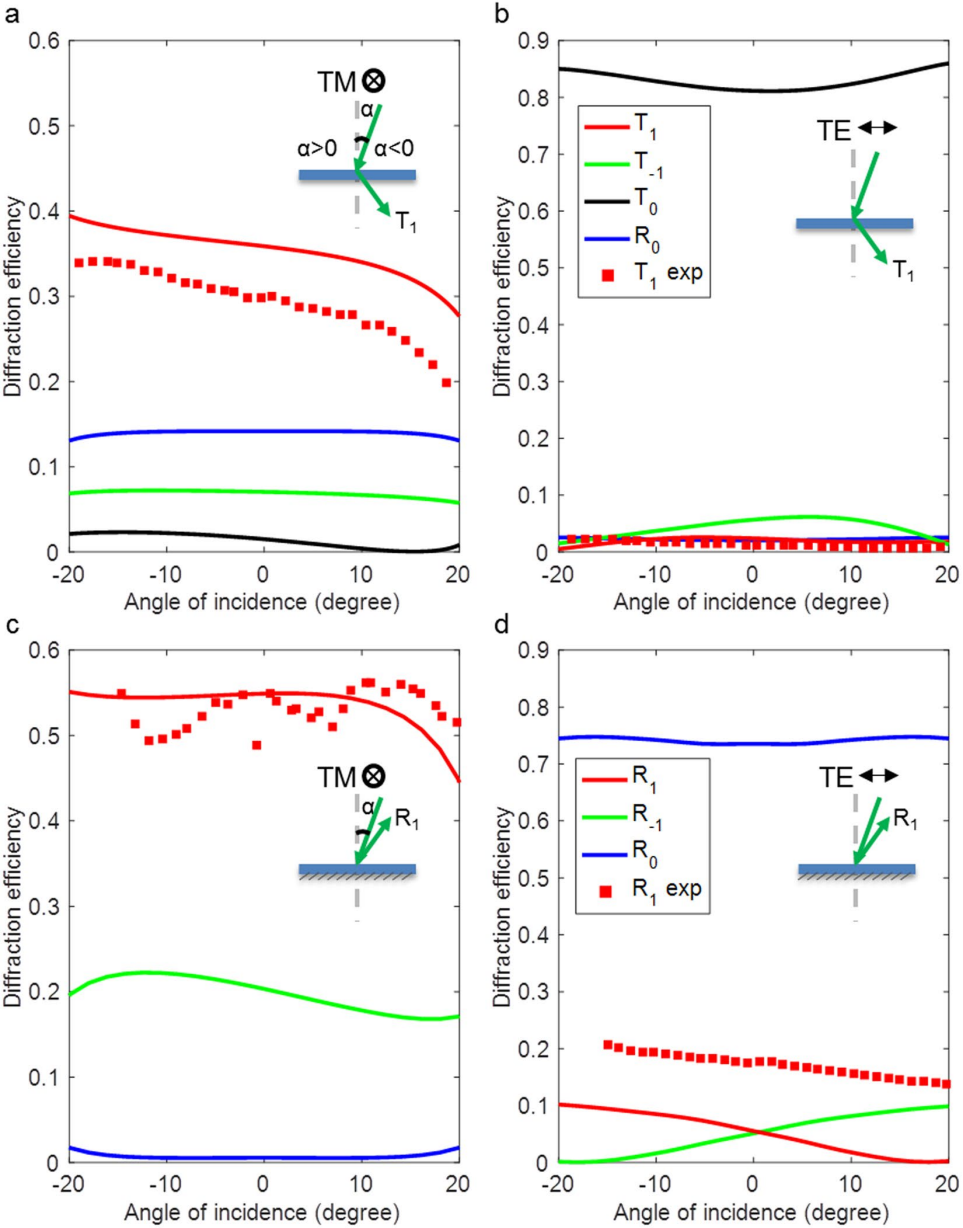
Transmittance and reflectance of TM and TE waves for metasurfaces working in transmission and reflection mode respectively at a wavelength of 520 nm. Diffraction efficiency versus incidence angle for transmission mode metasurfaces under illumination with (**a**) TM polarization and (**b**) TE polarization respectively. The red lines represent the theoretical (continuous line) and experimental (solid squares) diffraction efficiency of the +1 transmitted order T_1_. The green solid line shows the diffraction efficiency of the −1 transmitted order T-_1_. The black solid line and the blue solid line represent the zeroth-order transmittance T_0_ and reflectance R_0_, respectively. Diffraction efficiency versus incidence angle for reflection mode metasurfaces under illumination with (**c**) TM polarization and (**d**) TE polarization respectively, where the red lines represent the theoretical (continuous line) and experimental (solid squares) diffraction efficiency of the +1 reflected order R_1_. The green solid line shows the diffraction efficiency of −1 reflected order R-_1_. The blue solid line represents the reflectance R_0_ respectively. The graphs plotted on a logarithmic scale are shown in Supplementary Fig. S7.

Figure 4 shows the transmittance and reflectance of transverse magnetic (TM) and transverse electric (TE) waves for metasurfaces working in transmission and reflection mode respectively at a wavelength of 520 nm. Figure 4a shows the diffraction efficiency versus angle of incidence for transmission mode metasurfaces at a wavelength of 520 nm with TM polarization. The theoretical prediction of diffraction efficiency of our metasurfaces is calculated by finite element simulations. The diffraction efficiency is defined by the ratio of the power of light that has been steered into the first order of diffraction to the total power that is incident on the metasurface. For TM polarization, it is shown that the diffraction efficiency of the +1 transmitted order T_1_ is approximately 35% over a wide range of incidence angles with a variation of 10% across the inclusive angular range. The diffraction efficiency of the asymmetric grating across the entire angular range of incidence angles is relatively uniform. In comparison, for a grating composed of a single Si nanobeam within one unit cell, the diffraction efficiency dramatically changes when the incidence angle is off-normal (see Supplementary Fig. S5). Therefore the metasurface with an asymmetric grating design is essential to maintain the uniform efficiency for varying incidence angles. The diffraction efficiency for the −1 transmitted order T-_1_ is approximately 8%, which is much lower than the +1 transmitted order T_1_. It is shown that the proposed metasurfaces with tilt phase profiles can steer the light in the desired direction and suppress the diffraction into the −1 transmitted order. The efficiency of the zeroth-order transmittance is lower than 5%, while 15% of the light is reflected which can be potentially suppressed by adding an antireflective coating.

Here we have designed transmission mode metasurfaces with reasonable diffraction efficiency based on amorphous Si, which facilitate lower absorptive losses in the visible spectrum as compared to metallic metasurfaces. However, the absorption of amorphous Si in the visible spectrum limits further improvement in diffraction efficiency. For example, the Si nanobeams in the proposed transmission mode metasurface absorb 40% of incident light due to the material absorption of amorphous Si. An alternative material, such as crystalline Si, which has a high refractive index but is less absorptive at visible wavelengths can boost the diffraction efficiency to 60% at a wavelength of 520 nm (see Supplementary Fig. S6). However, the deposition of a thin-film of crystalline Si requires a complex fabrication process, so we only show it in simulation. We have also considered other materials which are more transparent at visible wavelengths but have lower refractive indexes. We have designed a transmission mode metasurface based on silicon nitride, as shown in Supplementary Fig. S6. When using silicon nitride, it is challenging to maintain the uniform diffraction efficiency across the entire range of incidence angles. Although 60% diffraction efficiency can be achieved at normal incidence, its diffraction efficiency drops to 30% at tilted angles of incidence, which is problematic in imaging and display systems. Additionally, due to the lower refractive index, the thickness and high aspect ratio of nanostructures based on Si_3_N_4_ are much larger than the one designed for Si, which make their fabrication more challenging.

The diffraction efficiencies of the fabricated samples were characterized by a custom-built goniometer. The experimental results of the transmission mode metasurface presented in Fig. 4a agree with modeling results. The experimental plots take into account calibrated through-prism absorption and the Fresnel losses at prism-air interfaces. The metasurface exhibits a relatively flat diffraction efficiency over a large range of incidence angles, exhibiting nearly 30% diffraction efficiency. For incidence angles larger than 25 degrees, when the deflection angles of the +1 transmitted order are larger than 90°, the diffraction efficiency falls to zero because the diffracted light becomes evanescent.

The transmittance and reflectance of metasurfaces under illumination with transverse electric (TE) polarization (with the electric field perpendicular to the nanobeams) is shown in Fig. 4b. It is shown that the diffraction efficiency of the +1 transmitted order is theoretically predicted and measured to be <2% over the same angular range with a polarization extinction ratio (the ratio between the diffraction efficiency in two orthogonal polarizations) larger than 15:1. The zeroth-order transmittance of TE waves is larger than 80% showing that the majority of the light waves are transmitting through the metasurface undisturbed. Therefore, this metasurface containing an asymmetric grating could potentially serve as a polarization beam splitter, which otherwise cannot be achieved by using a conventional blazed grating. The polarization selective response could be utilized in advanced imaging and display systems. The metasurface functioning in transmission mode offers the advantage of easy integration into an optical path.

Figure 4c shows the diffraction efficiency of the reflection mode metasurface across angles of incidence under illumination of TM polarized illumination at a wavelength of 520 nm. It shows a larger diffraction efficiency compared to the transmission mode metasurface. The reflection mode metasurface was also measured for green light at 520 nm. The diffraction efficiency of this sample closely matches the model reaching ~55% across a wide angular range for the designed linear polarization orientation. The small oscillation seen in the data is the result of a slight index mismatch between the index-matching fluid used to couple light between the sample substrate and the prism, which creates a weak Fabry-Perot effect. The thinner metasurface based on Si in reflection mode results in lower absorption (~25%) thanks to lower material absorption. Also, the thinner metasurface exhibits an aspect ratio of thickness to width smaller than 1:1, which is beneficial for nanofabrication. For orthogonal transverse electric (TE) polarization as shown in Fig. 4d, the diffraction efficiency of TE waves is expected to be much lower than TM polarization with a polarization extinction ratio about 10:1 upon normal incidence. The measured diffraction efficiency of TE waves is not as low as the theoretical prediction, which we attribute to fabrication inaccuracies and a small amount of birefringence in the PMMA layer.

**Figure 5:**
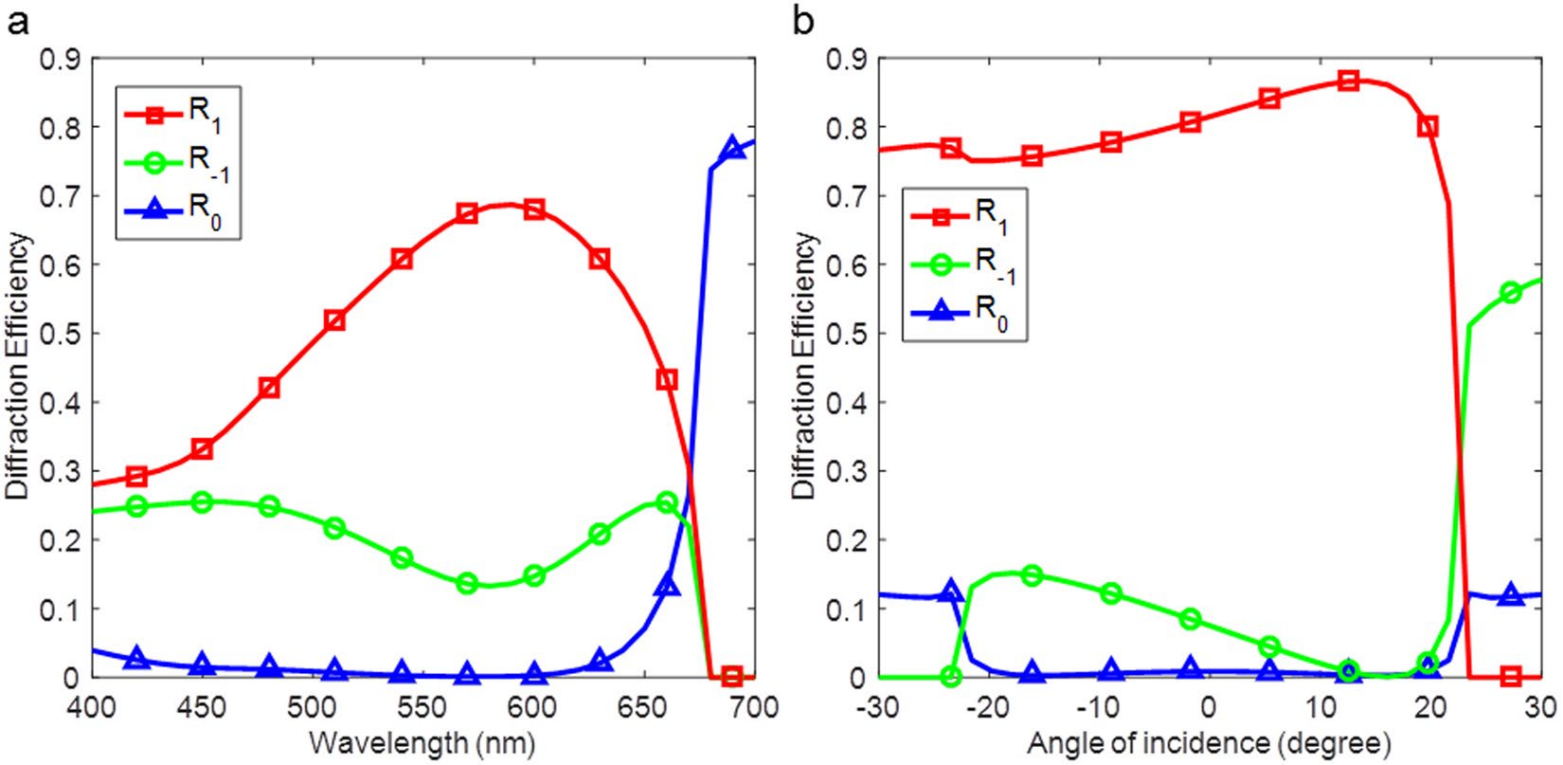
Broadband operation and optimal design for red wavelength. (**a**) Simulated spectra of diffraction efficiency of TM waves for the reflection mode metasurface at normal incidence, in the 400 to 700 nm wavelength range. The nanostructures of this metasurface were described in Fig. 2. (**b**) Simulated diffraction efficiency of TM waves versus incidence angle for different reflection mode metasurface at wavelength of 638 nm. This metasurface is optimized to efficiently diffract light over a wide range of incidence angles at wavelength of 638 nm. The nanobeams optimized for red wavelength have a thickness of 35 nm and widths of 30 nm and 90 nm, with an edge-to-edge gap of 60 nm. The red solid line with square markers shows the diffraction efficiency of the +1 reflected order, the green line with circle markers represents the diffraction efficiency of the −1 reflected order and the blue line with triangular markers shows the zeroth order reflectance.

The designed metasurfaces are capable of diffracting light to large deflection angles over a broad wavelength range at normal incidence. We calculate the diffraction efficiency of the +1 reflected diffraction order of reflection mode metasurface, as discussed in Figs 2 and 4c. The simulated diffraction efficiency spectra of TM waves for metasurfaces with normally incident light in the wavelength range from 400 to 700 nm are shown in Fig. 5a. It is shown that the diffraction efficiency is larger than 30% over a spectral bandwidth of 200 nm. For wavelengths longer than 650 nm, the diffraction efficiency dramatically decreased to zero because the corresponding deflection angle is larger than 90° and the light waves become evanescent. The absorption due to material loss limits the overall diffraction efficiency. For longer wavelengths where Si has much lower absorption, the metasurfaces can achieve a higher diffraction efficiency. For example, we have designed another reflection mode metasurface that can efficiently steer light to large deflection angles at a wavelength of 638 nm. The nanobeams optimized for red wavelength have a thickness of 35 nm and widths of 30 nm and 90 nm with an edge-to-edge gap of 60 nm. As shown modeled in Fig. 5b, it can achieve diffraction efficiency of TM waves as high as 80% over a large range of acceptance angles.

## Discussions

In conclusion, we have demonstrated two different types of metasurfaces for ultra-compact beam manipulation at visible wavelengths, which operate in transmission and reflection mode, respectively. Both metasurfaces are capable of steering light to large deflection angles with high diffraction efficiency over a broad range of incidence angles. The transmission mode metasurface can be more easily integrated into the optical path of an optical system, while the reflected metasurface consists of thinner Si nanobeams for easier nanofabrication and shows a higher diffraction efficiency. The fabrication of Si-based metasurfaces can capitalize on the extensive and mature fabrication technology that is available for this material. The demonstrated metasurfaces open up new opportunities in the field of spectroscopy, advanced imaging and display.

## Methods

### Nanofabrication

The fabrication process of the transmission and reflection mode metasurfaces begins with the deposition of a thin silicon film on a transparent substrate by plasma-enhanced chemical vapor deposition (PECVD). The thickness of the deposited silicon film is varied between 20 and 120 nm depending to the design of the metasurface. The nanostructures of metasurfaces are defined on negative Hydrogen Silsesquioxane (HSQ) resist by using electron beam lithography (EBL). The nanostructures are transferred into silicon film by ICP plasma etching with an HBr-based process. The remaining HSQ mask is left on top of the silicon lines. The reflection mode metasurface samples require additional steps. After patterning the Si nanostructures, a layer of Poly(methyl methacrylate) (PMMA) is spun over nanostructures at different speeds to control the thickness of the spacer. As a final step, an Ag(100 nm)/Au(50 nm) reflective bilayer is deposited on the spacer.

### Optical characterization

Measurements of the transmittance and reflectance of metasurfaces were made using a motorized custom-built goniometer, which uses a dovetail prism and a fiber-based laser diode delivery system. This system allows measuring the angular response of the optical elements in any state of polarization at a given wavelength. The sample is placed on the top of the prism, and a particular polarization is selected through a combination of rotating half and quarter-wave plates. The beam incident on the sample exhibits a spot size of 100 μm and stays centered on the same location throughout the angular range. This was monitored by a Point Gray Research camera and Edmund Optics long working distance microscope objective. To minimize Fresnel losses, we use an appropriate refractive index-matching fluid, while the sample alignment is achieved by using a x-y-z precision differential micrometer stage. The intensities of incident and diffracted beams are simultaneously measured by silicon photodiodes as the goniometer scans across the preset angular range.

## Electronic supplementary material

Supplementary Information
