# Supplementary material for: Optical metasurfaces for high angle steering at visible wavelengths
Source: Sci Rep. 2017 May 23;7:2286. doi: 10.1038/s41598-017-02167-4 (PMC5442109; doi:10.1038/s41598-017-02167-4)
Supplement: Supplementary file 1 — Supplementary Information [file 41598_2017_2167_MOESM1_ESM.pdf]

Supplementary Information

Optical metasurfaces for high angle steering at visible wavelengths

Dianmin Lin[1*], Mauro Melli[1], Evgeni Poliakov[1], Pierre St. Hilaire[1], Scott Dhuey[3], Christophe Peroz[1], Stefano Cabrini[3], Mark Brongersma[2] and Michael Klug[1]

[1] Magic Leap Inc., Plantation, FL 33322, USA
[2] Geballe Laboratory for Advanced Materials, Stanford University, Stanford, CA 94305, USA
[3] The Molecular Foundry, Lawrence Berkeley National Laboratory, Berkeley, CA 94720, USA
* To whom correspondence should be addressed. E-mail: dlin@magicleap.com.

S1. Angular optical response of subwavelength nanobeam array

The optical response of Si nanobeam arrays with subwavelength periodicity of 125 nm at normal incidence is shown in Fig.1. It has been shown that the phase delay can be tuned by changing the width of the nanobeams. Fig. S1 shows the phase delay and amplitude of light transmitted through the nanombeam array at different incidence angles. It can be seen that the optical response of the nanobeam array is very uniform across the angular range.

The optical response of the metasurface can largely be understood by understanding the scattering properties of the individual, constituent Si nanobeams. Each nanobeam can be considered as a slab waveguide that has been truncated in the propagation direction. The acceptance angle of a slab waveguide is determined by the refractive index of its core material and cladding material. Here, each nanobeam has a large acceptance angle thanks to the high refractive index contrast between silicon and air. In addition, the silicon nanobeams are subwavelength in thickness, which reduces the shadow effect from neighboring antennas.

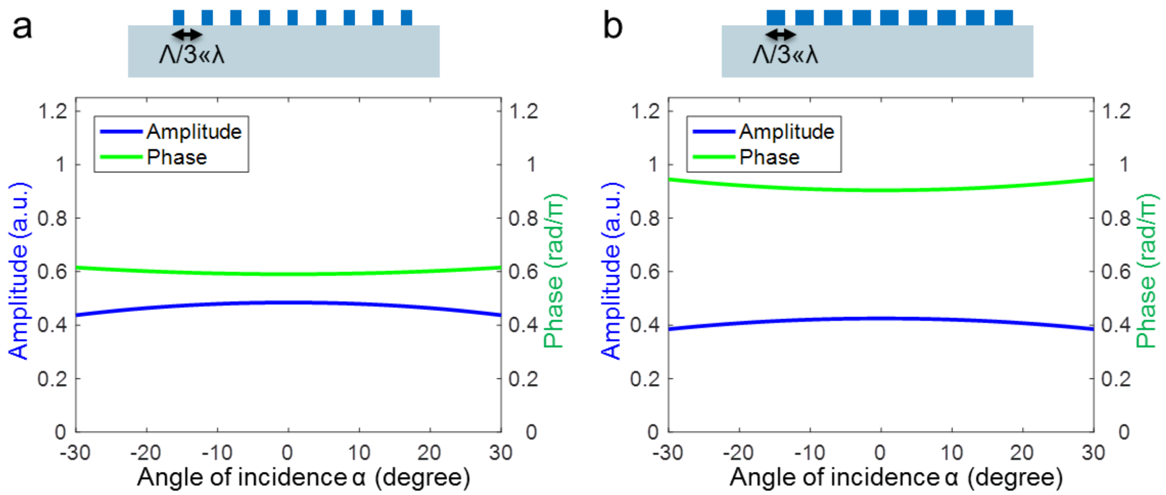

Figure S1. Variation of the phase delay and amplitude of the transmitted light versus angle of incidence for an array of Si nanobeams with periodicity of 125 nm, and width of (a) 30 nm and (b) 55 nm respectively.

S2. Deflection Angle

The deflection angle θ varies with incident angle α and wavelength, which follows from k-vector matching and the grating equation:

$$k_2 = k_{inc} + m\frac{2\pi}{\Lambda} \tag{S1}$$

$$\frac{\sin\theta}{n\lambda} = \frac{\sin\alpha}{\lambda} + \frac{m}{\Lambda} \tag{S2}$$

$$\Lambda \sin\theta = \Lambda\, n \sin\alpha + n\, m\lambda \tag{S3}$$

where Λ is the grating periodicity, λ is the free space wavelength, n is the refractive index of the substrate, and m is the diffraction order. The variation of deflection angle θ versus incidence angle α at a wavelength of 520 nm is plotted in Fig. S2. The diffraction efficiency of the metasurface for TM polarization is also plotted in the figure on the right axis, showing that the diffraction efficiency remains uniform across a wide angular range.

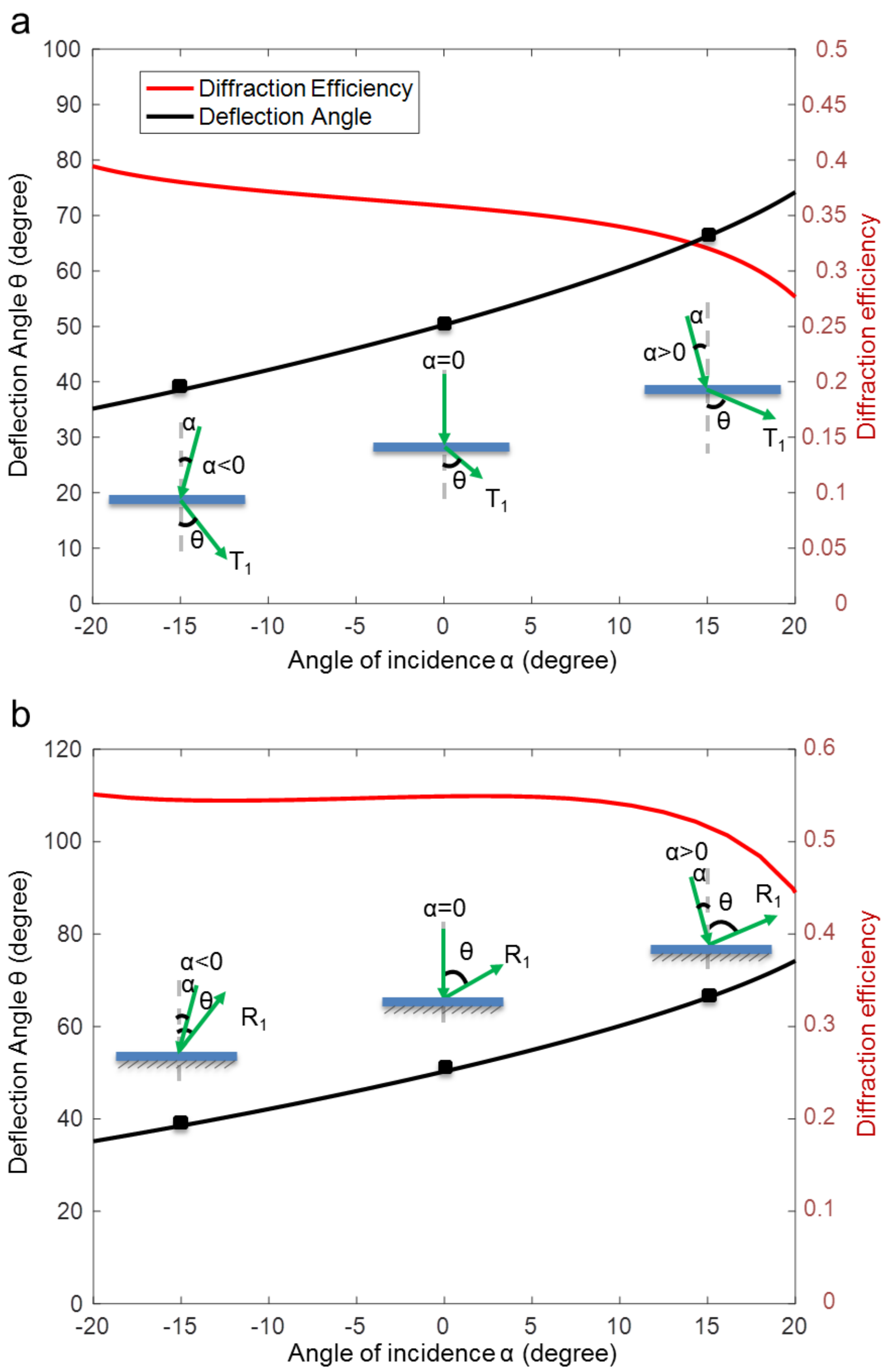

Figure S2. Deflection angle θ and diffraction efficiency of TM polarization versus incidence angle α at a wavelength of 520 nm for (a) a transmission mode metasurface and (b) a reflection mode metasurface.

Figure S3 shows the deflection angle θ and diffraction efficiency versus wavelength upon normal incidence for the reflection mode metasurface. The deflection angle θ will increase with wavelength, as shown on the left axis in Fig.S3.

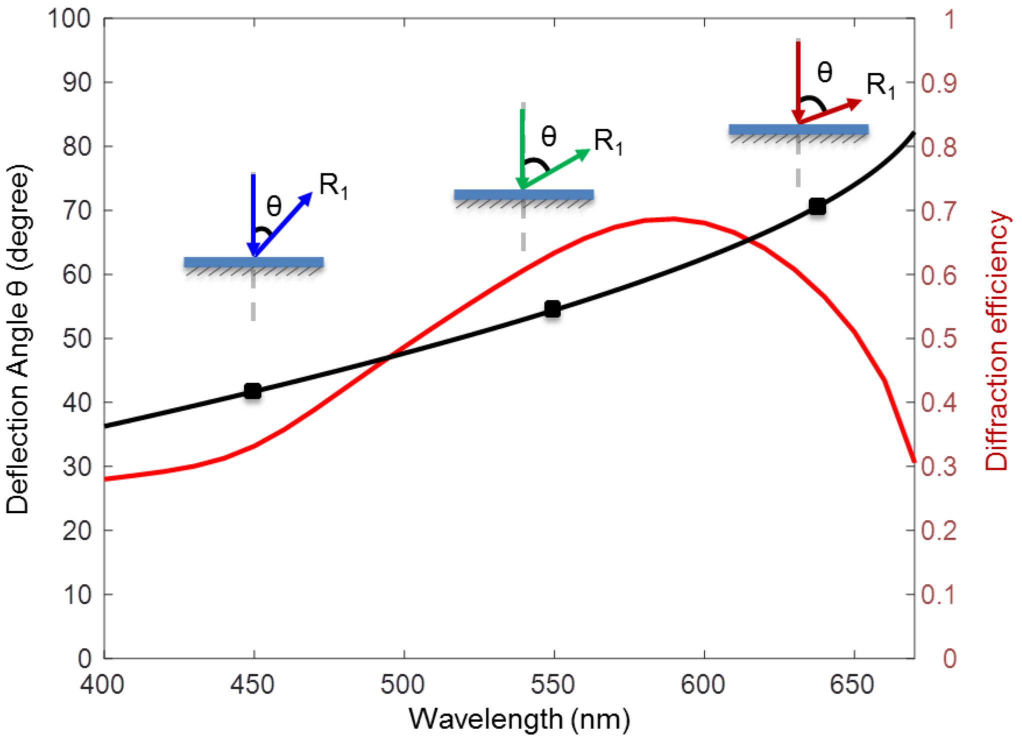

Figure S3. Deflection angle θ and diffraction efficiency versus wavelength upon normal incidence for reflection mode metasurfaces.

S3. Building blocks of reflection mode metasurface

The phase delay of the transmitted light through the array of nanobeams can be controlled by the thickness and width of the nanobeams. In reflection mode, the light interacts with the nanobeams twice (once incident and again after reflection off the back mirror), which gives rise to a larger phase change. Figure S4a shows the variation of the phase and amplitude of light reflected from metasurfaces versus the width of nanobeams, where the nanobeams are 25-nm-thick and spaced by 95 nm. It is shown that a 25-nm-thick Si nanostructure array in reflection mode is capable of achieving the same amount of phase delay as a 75-nm-thick Si nanostructure array in transmission mode. In addition, the thinner metasurface based on Si in reflection mode results in lower absorption due to lower material absorption. It is shown that amplitude of the light wave in the reflection mode metasurface is larger compared to the transmission mode metasurface, which leads to a higher diffraction efficiency.

Figure S4b shows the wavefront of light waves reflected from metasurface, without the Si nanobeams and with a subwavelength periodic array of nanobeams of 30 nm and 60 nm widths. The incident light is subtracted out in the field plot, showing only the reflected light. It is shown that the retardation of wavefront of the reflected light waves can be modulated by engineering the size of the nanobeams. These subwavelength nanobeams will serve as the building block of a TM-polarized reflection mode metasurface for high angle light steering at wavelength of 520 nm.

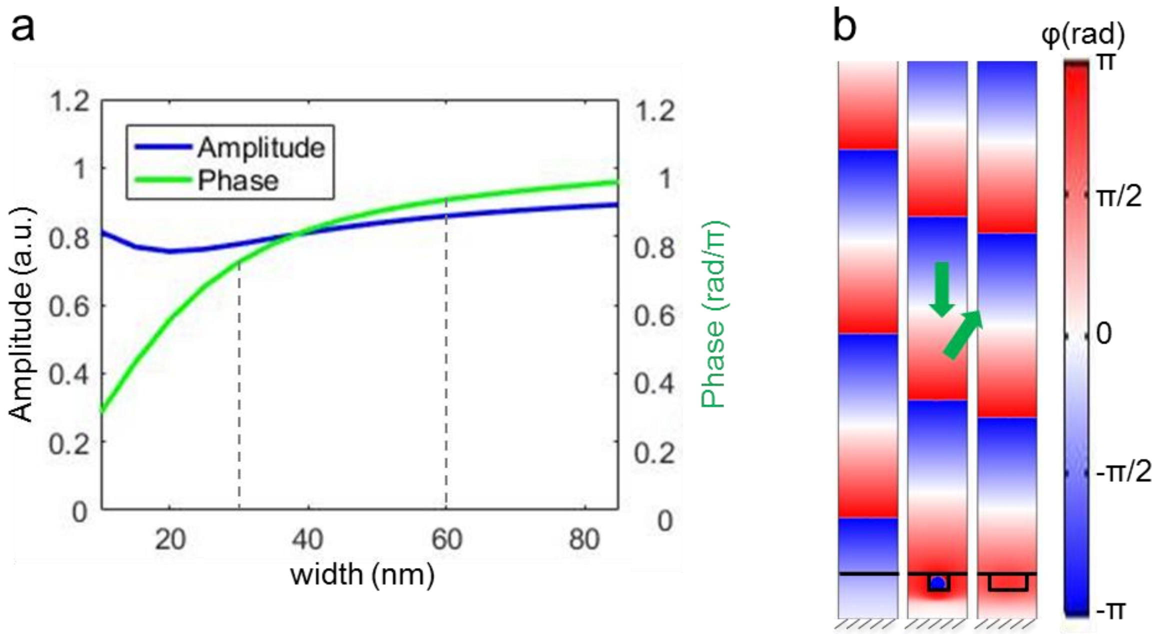

Figure S4. Building blocks of TM-polarized reflection mode metasurface at a 520-nm wavelength. (a) The variation of the phase delay and amplitude of the reflected light versus the width of the Si nanobeams of the subwavelength periodic arrays. (b) Phase wavefront of light waves reflected from metasurface, without the Si nanobeams and with a subwavelength periodic array of nanobeams of 30 nm and 60 nm widths.

S4. Diffraction efficiency of a binary grating

The diffraction efficiency of an asymmetric grating across the incidence angular range is quite uniform. In comparison, for a grating composed of a single Si nanobeam within one unit cell, the diffraction efficiency dramatically changes at off-normal incidence angles, as shown in Fig. S5.

We have investigated the diffraction properties of a binary grating, which is composed of a nanobeam array with width of 30 nm, thickness of 75nm, and periodicity 380nm. The diffraction efficiency of the binary grating with narrower nanobeams is plotted Fig. S5a. For a binary grating, the diffraction efficiency of +1 and -1 orders are equal to 23% at normal incidence, because the structure is symmetric. When the incidence angle is off-normal, gradually increasing to 20 degrees, the diffraction efficiency of the +1 order dramatically decreases while the energy in the -1 order increases. The diffraction efficiency of the +1 and -1 orders are complementary across the angular range of incidence angles due to the mirror symmetry of the grating. The diffraction efficiency of a binary grating with wider nanobeams having widths of 55 nm is plotted Fig. S5b. Note that higher proportion of the incident light is absorbed, due to the larger size of the nanobeams.

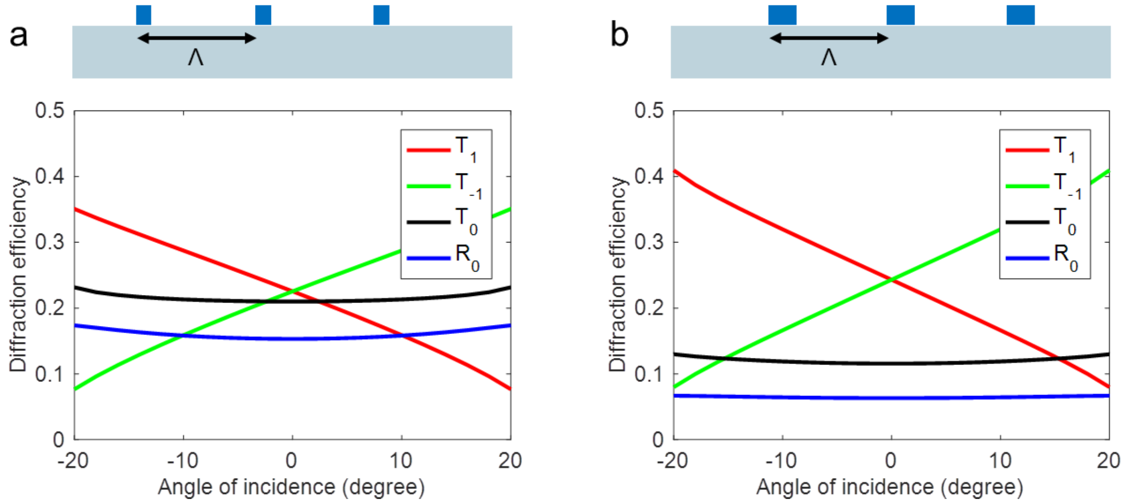

Figure S5. The diffraction efficiency of a binary grating of nanobeam array, with a thickness of 75nm, periodicity of 380nm, and width of (a) 30 nm and (b) 55 nm, respectively.

S5. Other materials

We have also investigated other materials that exhibit low attenuation at visible wavelengths. An alternative material, such as crystalline Si, which has high refractive index but is less absorptive than amorphous Si in the visible spectrum, can boost the diffraction efficiency to 60% at a wavelength of 520 nm, as shown in supplementary Fig. S6a. However, the deposition of quality crystalline Si thin-films requires advanced fabrication processes, so we only demonstrate it in simulation. We have also considered other materials which are transparent at visible wavelengths but exhibit lower refractive index than silicon. An example based on silicon nitride is shown in supplementary Fig.S6b. However, it is challenging to maintain uniform diffraction efficiency across the whole range of incidence angles with lower index materials. Although it can achieve 60% diffraction efficiency at normal incidence, its diffraction efficiency drops down to 30% at

tilted angles of incidence, which is problematic in imaging and display applications. Additionally, due to the lower refractive index, the thickness and high aspect ratio of nanostructures based on Si_3N_4 are much larger than the one designed for Si, which makes their fabrication more challenging.

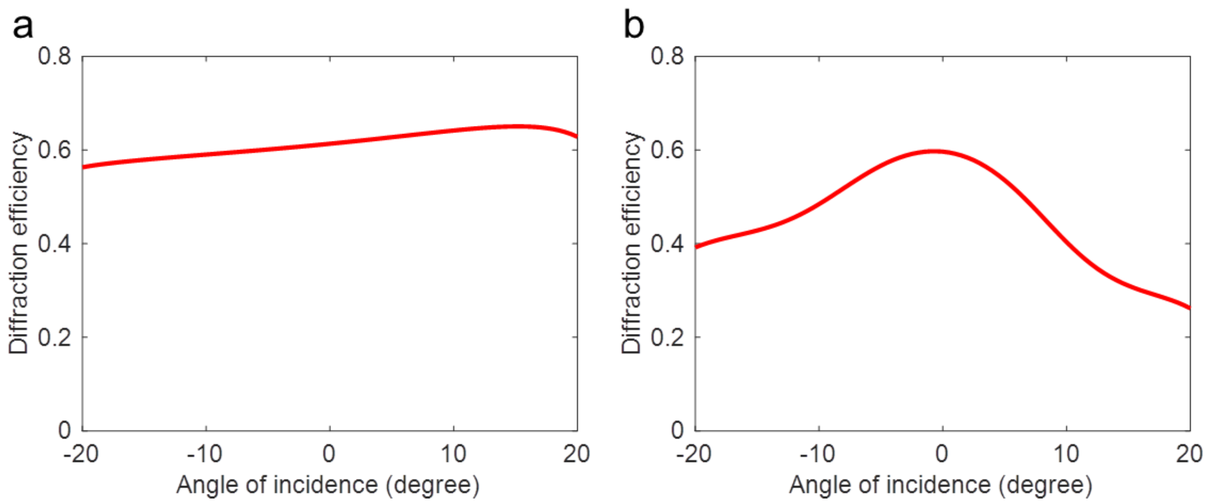

Figure S6. The diffraction efficiency of metasurfaces based on (a) c-Si and (b) silicon nitride respectively.

S6. Transmittance and reflectance plotted on a logarithmic scale

We have shown the transmittance and reflectance of metasurfaces under illumination with both transverse magnetic (TM) and transverse electric (TE) polarization in Fig. 4. The same results are plotted on a logarithmic scale in Fig. S7 as well, showing more clearly the small values.

Note that the experimental data for angle of incidence from -16° to -20° in Fig. S7c and Fig. S7d are absent due to the limitation of the measurement tool. For the reflection mode metasurface, it is difficult to measure the power of the +1 reflected diffraction order R_1 at large angles of incidence from -16° to -20°. For example, the direction of diffracted beam R_1 is nearly coincident with that of the incident beam when the incident angle is -20°, as shown in the inset in Fig. S7c. In this case, it is difficult to separate the two beams and measure the intensity of the diffracted beam, unless we use a beam splitter, which adds additional complexity and inaccuracies into the current measurement tool.

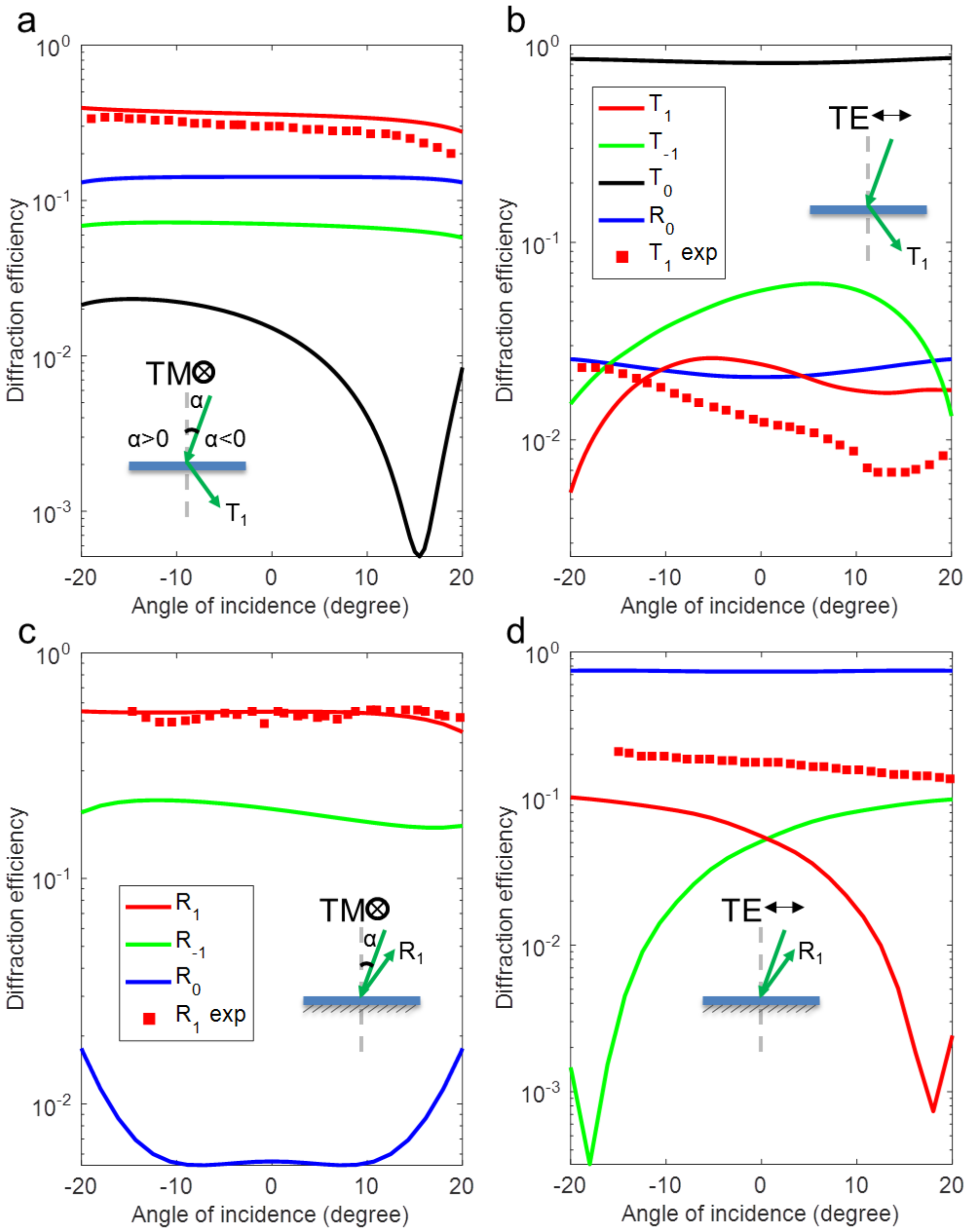

Figure S7. Results in Figure 4 plotted on a logarithmic scale. Transmittance and reflectance of TM and TE waves for metasurfaces working in transmission and reflection mode respectively at a wavelength of 520 nm. Diffraction efficiency versus incidence angle for transmission mode metasurfaces under illumination with (a) TM polarization and (b) TE polarization respectively. The red lines represent the theoretical (continuous line) and experimental (solid squares) diffraction efficiency of the +1 transmitted order T1. The green solid line shows the diffraction efficiency of the -1 transmitted order T-1. The black solid line and the blue solid line represent the zeroth-order transmittance T0 and reflectance R0, respectively. Diffraction efficiency versus incidence angle for reflection mode metasurfaces under illumination with (c) TM polarization and (d) TE polarization respectively, where the red lines represent the theoretical (continuous line) and experimental (solid squares) diffraction efficiency of the +1 reflected order R1. The green solid line shows the diffraction efficiency of -1 reflected order R-1. The blue solid line represents the reflectance R0 respectively.
